# Supplementary material for: Modeling glioblastoma heterogeneity as a dynamic network of cell states
Source: Mol Syst Biol. 2021 Sep 16;17(9):e10105. doi: 10.15252/msb.202010105 (PMC8444284; doi:10.15252/msb.202010105)
Supplement: Supplementary file 6 — Source Data for Figure 5 [file MSB-17-e10105-s004.zip › Figure5A_sourcedata/GSEA_3071/hallmarks_stateV.GseaPreranked.1623742546329/HALLMARK_INTERFERON_ALPHA_RESPONSE.html]

Details for gene set HALLMARK\_INTERFERON\_ALPHA\_RESPONSE[GSEA]

|  || Dataset | state43071 |
| Phenotype | NoPhenotypeAvailable |
| Upregulated in class | na\_neg |
| GeneSet | HALLMARK\_INTERFERON\_ALPHA\_RESPONSE |
| Enrichment Score (ES) | -0.49217847 |
| Normalized Enrichment Score (NES) | -2.163224 |
| Nominal p-value | 0.0 |
| FDR q-value | 2.863095E-4 |
| FWER p-Value | 0.002 |
Table: GSEA Results Summary

  

Fig 1: Enrichment plot: HALLMARK\_INTERFERON\_ALPHA\_RESPONSE      
 Profile of the Running ES Score & Positions of GeneSet Members on the Rank Ordered List

  

| PROBE | GENE SYMBOL | GENE\_TITLE | RANK IN GENE LIST | RANK METRIC SCORE | RUNNING ES | CORE ENRICHMENT || 1 | GMPR |  |  | 1249 | 0.546 | -0.2056 | No |
| 2 | CNP |  |  | 1879 | 0.406 | -0.3016 | No |
| 3 | USP18 |  |  | 1885 | 0.406 | -0.2790 | No |
| 4 | IL7 |  |  | 2500 | 0.322 | -0.3770 | No |
| 5 | CD47 |  |  | 2503 | 0.322 | -0.3586 | No |
| 6 | PARP9 |  |  | 2856 | 0.284 | -0.4091 | No |
| 7 | PSME1 |  |  | 3202 | -0.251 | -0.4600 | No |
| 8 | NCOA7 |  |  | 3301 | -0.266 | -0.4632 | No |
| 9 | DDX60 |  |  | 3304 | -0.266 | -0.4481 | No |
| 10 | TRIM5 |  |  | 3328 | -0.271 | -0.4367 | No |
| 11 | PARP12 |  |  | 3343 | -0.274 | -0.4235 | No |
| 12 | PNPT1 |  |  | 3513 | -0.302 | -0.4381 | No |
| 13 | IFITM2 |  |  | 3585 | -0.312 | -0.4334 | No |
| 14 | CASP1 |  |  | 3648 | -0.323 | -0.4265 | No |
| 15 | NUB1 |  |  | 3659 | -0.324 | -0.4096 | No |
| 16 | RIPK2 |  |  | 3867 | -0.362 | -0.4279 | No |
| 17 | IFIT3 |  |  | 3874 | -0.362 | -0.4080 | No |
| 18 | PSMB8 |  |  | 4318 | -0.477 | -0.4645 | Yes |
| 19 | B2M |  |  | 4341 | -0.485 | -0.4405 | Yes |
| 20 | IL15 |  |  | 4346 | -0.486 | -0.4131 | Yes |
| 21 | IFI44L |  |  | 4387 | -0.497 | -0.3918 | Yes |
| 22 | PSME2 |  |  | 4422 | -0.508 | -0.3688 | Yes |
| 23 | IFITM3 |  |  | 4424 | -0.509 | -0.3394 | Yes |
| 24 | PSMB9 |  |  | 4503 | -0.543 | -0.3227 | Yes |
| 25 | ISG15 |  |  | 4586 | -0.579 | -0.3047 | Yes |
| 26 | TXNIP |  |  | 4618 | -0.604 | -0.2755 | Yes |
| 27 | SAMD9L |  |  | 4638 | -0.613 | -0.2436 | Yes |
| 28 | PSMA3 |  |  | 4650 | -0.618 | -0.2098 | Yes |
| 29 | EPSTI1 |  |  | 4742 | -0.672 | -0.1880 | Yes |
| 30 | SAMD9 |  |  | 4850 | -0.755 | -0.1645 | Yes |
| 31 | ELF1 |  |  | 4927 | -0.841 | -0.1301 | Yes |
| 32 | PROCR |  |  | 5097 | -1.139 | -0.0961 | Yes |
| 33 | NMI |  |  | 5098 | -1.142 | -0.0298 | Yes |
| 34 | IRF1 |  |  | 5100 | -1.155 | 0.0371 | Yes |
Table: GSEA details [plain text format]

  

Fig 2: HALLMARK\_INTERFERON\_ALPHA\_RESPONSE: Random ES distribution      
 Gene set null distribution of ES for **HALLMARK\_INTERFERON\_ALPHA\_RESPONSE**
